# Behçet’s Disease Uveitis

**DOI:** 10.3390/jcm12113648

**Published:** 2023-05-24

**Authors:** Morgane Joubert, Anne-Claire Desbois, Fanny Domont, Amine Ghembaza, Alexandre Le Joncour, Adrien Mirouse, Georgina Maalouf, Mathilde Leclercq, Sarah Touhami, Patrice Cacoub, Bahram Bodaghi, David Saadoun

**Affiliations:** 1 AP-HP. Centre de Référence des Maladies Auto-Immunes Systémiques Rares, Centre de Référence des Maladies Auto-Inflammatoires et de l’Amylose Inflammatoire, F-75013 Paris, France; 2 CHU Rouen, Internal Medicine Department, F-76000 Rouen, France; 3 Department of Ophthalmology, Pitié-Salpêtrière University Hospital, Sorbonne Universités, F-75013 Paris, France; 4 Institut National de la Santé Et de la Recherche Médicale (INSERM), Unité Médical de Recherche (UMR)S 59, F-75013 Paris, France

**Keywords:** Behçet’s disease, uveitis, vasculitis, biotherapies, anti TNF-α agents

## Abstract

Uveitis in Behçet’s disease (BD) is frequent (40% of cases) and is a major cause of morbidity. The age of onset of uveitis is between 20 and 30 years. Ocular involvement includes anterior, posterior, or panuveitis. Uveitis may be the first sign of the disease in 20% of cases or it may appear 2 or 3 years after the first symptoms. Panuveitis is the most common presentation and is more commonly found in men. Bilateralization usually occurs on average 2 years after the first symptoms. The estimated risk of blindness at 5 years is 10–15%. BD uveitis has several ophthalmological features that distinguish it from other uveitis. The main goals in the management of patients are the rapid resolution of intraocular inflammation, the prevention of recurrent attacks, the achievement of complete remission, and the preservation of vision. Biologic therapies have changed the management of intraocular inflammation. The aim of this review is to provide an update to a previous article by our team on pathogenesis, diagnostic approaches, and the therapeutic strategy of BD uveitis.

## 1. Introduction

**Figure 1:**
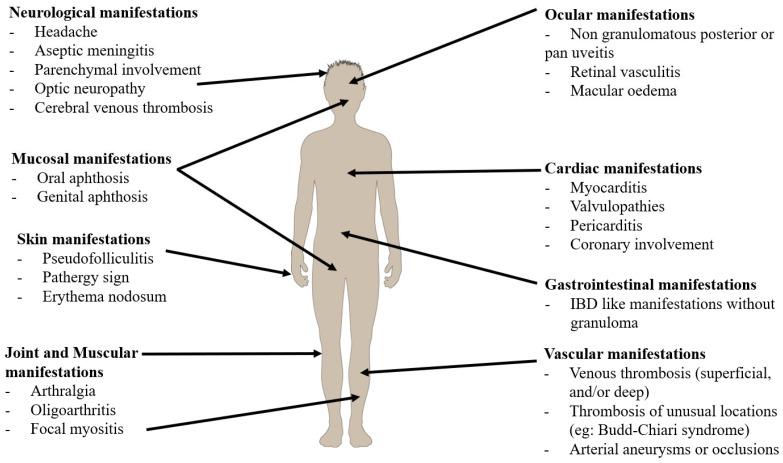
Summary of clinical manifestations in Behçet’s disease (extracted from French recommendations for the management of Behçet’s disease. Kone-Paut, I. et al. (2018) [7]).

Behçet’s disease (BD) is a systemic vasculitis (Figure 1) at the crossroads between autoimmune and autoinflammatory diseases [1,2]. Uveitis is one of the most severe complications [3] and progress in biologic therapy has transformed the visual outcomes [4]. There are current gaps in knowledge and unmet clinical needs in BD-associated uveitis. The current issues in uveitis associated with BD are to better define ophthalmological criteria, and diagnostic algorithms, with an improvement of ocular multimodal imaging advances in eye imaging, to shorten the delay of induction therapy and to optimize the use of biological therapies. The major therapeutic challenge is to treat as early as possible with targeted treatment to limit the visual sequelae and optimize the inflammation control. Our aim was to provide an up-to-date [5,6] review on the biomarkers, diagnostic approach, and therapeutic strategy in BD uveitis.

## 2. Epidemiology and Pathophysiology

Epidemiology shows large geographic variations in BD frequency, with prevalence rates per 100,000 inhabitants of 20–420 for Turkey, 1.5–15.9 for southern Europe, and 0.3–4.9 for northern Europe [8]. Interestingly, ethnic disparities persist among higher-prevalence migrants or their descendants living in lower-prevalence areas [9]. Familial cases account for less than 5% [10]. The incidence in patients under 25 years old is higher [11] and young males have the worst prognosis [12,13]. BD uveitis occurs in 50% to 60% of patients [1,14].

The involvement of innate immunity and the vascular infiltration of activated neutrophils has been widely reported in the pathogenesis of BD [15,16]. The inflammation observed in the pathergy test suggests the activation of pattern recognition receptors (PRRs) by DAMPs and PAMPs [17]. However, PRRs gave an unaltered IL-1α, IL-6, TNF-α, IFN-α, and IL-18 response to stimulation. Increased expression and altered activation of toll-like receptors (TLRs) was described in BD [18]. TLR activation results via transcription factor NF-κB in the production of pro-inflammatory cytokines, such as TNF-α [17]. Reactive oxygen species (ROS), produced at the site of inflammation, cause endothelial dysfunction and tissue damage [19,20] and induce NETosis. Natural killer (NK) cells are increased in peripheral blood and BD lesions during the active phases of the disease [21] and contribute to the initiation of the Th1 response [22]. Activated (γδ) T cells are increased in BD patients’ peripheral blood and accumulate at inflammatory sites [23,24]. Anti-endothelial cell antibodies (AECAs) have been described in BD [25,26,27] and could trigger inflammation through complement or antibody-dependent cell toxicity, causing vasculitis.

IL-6, TNFα, and CXCL10 were found to be significantly elevated in the aqueous humor (AH) of patients with BD uveitis, sarcoidosis, and toxoplasmosis uveitis as compared to non-inflammatory controls [28]. Another study aimed at investigating the potential markers for BD uveitis and uveitis associated with Vogt Koyanagi Harada (VKH) disease compared to healthy controls (HCs), showed that IL-6, CXCL10, G-CSF, and IFNγ were in higher concentrations in AH samples from both BD and VKH patients whereas IL-2, IL-8, IL-13, TNFα, eotaxin, and IL-1ra showed statistically significant higher concentrations only in AH samples from BD patients. The levels of IL-6, IL-8, CXCL10, G-CSF, IFNγ, TNFα, eotaxin, and IL-1ra correlated positively with leukocyte levels in the AH of BD [29]. Another study showed that IL-6 serum levels were elevated in BD patients in the active stages of the disease [30]. It has also been shown that the levels of IL-23, IL-17, and IFN-γ are elevated in BD patients with active uveitis, suggesting that the IL-23/IL-17 pathway together with IFN-γ is associated with the active intraocular inflammation in BD patients [31,32].

Accumulating evidence shows that the combination of certain genetic or epigenetic factors causes an imbalance in the regulation of the immune response leading to the development of BD uveitis. Carrying the HLA-B*51 allele confers a relative risk of developing BD of 5.8. In addition to HLA-B*51, genome-wide association studies have identified various other polymorphisms in immune-related genes (*ERAP-1*, *IL23R-IL12RB2*, *IL10*, *STAT4*, *CCR1-CCR3*, *KLRC4*, *TNFAIP3*, *FUT2*, *MICA*) [33,34], IL23R/C1orf141, STAT4, and ADO/ZNF365/EGR2 [35].

In summary, in BD uveitis pro-inflammatory cytokines such as Il-6, TNFα, ΙFNγ, and IL-1ra are increased in AH. IL-6 levels are increased in the vitreous body and their concentration is positively correlated with concentration of leucocytes and disease activity. In terms of genetics, the interaction between leucocytes and MHC class I, and polymorphism in immune-related genes, seems to play an important role. From these physiopathological findings derive the therapeutic management detailed in the rest of the article.

## 3. Prognosis of Behçet’s Disease Uveitis

BD uveitis is responsible for a large amount of blindness in high prevalence countries. In a French cohort of sight-threatening uveitis receiving biotherapies, BD was independently associated with the poorest visual outcome [36]. In a Turkish series, the 3-year visual acuity was 20/200 or worse in 27.6% of the eyes of patients treated in 1990–1994 and 12.9% of the eyes of those treated in 2000–2004; this trend was explained by an earlier use of conventional disease-modifying antirheumatic drugs (cDMARDS) and biologics [37]. In the most recent series, the blindness rate ranged between 11% and 25% [38].

## 4. Diagnosis of Behçet’s Disease Uveitis

### 4.1. Diagnosis of Systemic Behçet’s Disease

The diagnosis of BD is based on the presence of clinical diagnostic criteria, established by the International Study Group (ISG) in 1990 [39] and revised in 2013 (Table 1) [39].

### 4.2. Diagnosis of Uveitis Associated with Behçet’s Disease

#### 4.2.1. Ocular Clinical Presentations

Uveitis may the initial manifestation, reported in 6–20% of patients [13,40,41]. Extra-ophthalmological signs are often overlooked, and it is therefore crucial to define the ophthalmological criteria. Both the anterior and posterior segments can be affected but panuveitis is the most frequent presentation. In addition, intermediate uveitis in the form of isolated vitritis is more common in early BD than late BD [42]. Episcleritis, scleritis, conjunctival ulcers, keratitis, orbital inflammation, isolated optic neuritis, and extraocular muscle palsies has been described [13]. The average age of onset is 25 years. Bilateralization usually occurs on average 2 years after the disease’s onset.

Isolated anterior uveitis (AU) affects less than 10% of patients. It presents as a sudden acute onset, with ocular redness, periorbital pain, photophobia, and tearing. It is always non-granulomatous, associated with anterior chamber Tyndall, and may be complicated by posterior synechiae. Hypopyon reflects the severity. The recurrence of AU may be complicated by glaucoma. An ocular hypertonia might be the result of angle closure due to anterior synechiae or pupillary occlusion, inflammation, or local or systemic administration of steroids [38].

Posterior uveitis is the most frequent and the most severe. It can threaten the visual prognosis. Posterior involvement can be present by an isolated visual acuity decreasing or be asymptomatic [6]. It may present as hemorrhagic retinitis areas of a variable number and distribution, or white-yellowish. In the case of macular localization, it may be associated with the visual acuity decreasing. Vitreous involvement may limit access to the fundus. Retinal vasculitis is common and mostly venous, but can be arterial or both. BD vasculitis is likely occlusive [43]. These peripheral ischemic areas may be complicated by pre-retinal or papillary neovascularization, which may cause retinal or vitreous hemorrhage, retinal ischemia, neovascularization and secondary neovascular glaucoma. Macular oedema may occur and affect the visual prognosis. Complications caused by recurrent posterior inflammatory flares include retinal atrophy, vascular sclerosis, optic atrophy, neovascular glaucoma, and retinal detachment [3]. Macular holes have also been reported and have led changes involving the vitreo-macular interface [44]. Moreover, localized retinal nerve fiber layer defects not associated with a retinochoroidal scar in the absence of glaucoma could guide the diagnosis of BD uveitis. They are linked to past foci of retinitis, which are transient and resolve without scar formation, and so could be missed [45].

#### 4.2.2. Ocular Investigations

Fundus photography is simple and economic. It can document and monitor the grade of vitreous damage [46].

Fundus fluorescein angiography (FA) is the gold standard imaging modality for the diagnosis and monitoring of BD uveitis. FA is a mandatory tool for the assessment of inflammatory fundus conditions due to posterior uveitis; the leakage on FA identifies retinal vasculitis and is an important marker of BD uveitis activity [47]. The specific signs of inflammatory activity include increased tortuosity of retinal veins, staining of vessel walls, leakage from large and small retinal vessels, and from the optic disc. Fern-like capillary leakage is the most characteristic FA finding in BD uveitis and may be present even when the uveitis seems inactive. Even if FA is a challenging assessment to perform in daily care, it remains critical to monitor BD uveitis activity [48].

Optical coherence tomography (OCT) is a non-invasive tool used to diagnose and to monitor macular complications such as macular edema, retinal cysts, severe retinal serious detachment, epiretinal membranes, vitreomacular traction, foveal atrophy, and macular holes [49].

*Enhanced Depth Imaging OCT* (EDI-OCT) provides detailed and measurable images of the choroid [50]. Subfoveal choroidal thickness may reflect macular vasculitis or inflammation; its measurement may be a non-invasive tool to investigate macular inflammatory activity in BD uveitis [50]. However, it should be noted that a study has been published with conflicting results, showing no increase choroidal thickening during active BD uveitis [51].

The inability to image the entire retinal capillary system is a main restriction of FA. Optical coherence tomography angiography (OCTA) is a rapid, non-invasive diagnostic imaging technique that detects movement in blood vessels, without contrast injection and provides depth-resolved visualization of the retinal and choroidal vascularization [52]. OCTA has been shown to better visualize microvascular changes in the macular area, such as capillary dropout, increased foveal avascular zone, telangiectasias, shunts, and neovascularization zone, than FA in eyes with active BD uveitis. The deep capillary plexus appears to be more affected than the superficial capillary plexus [14,52,53].

#### 4.2.3. Strategy for Earlier Diagnosis of BD Uveitis

BD uveitis has several distinctive clinical features (Table 2). Recently, Tugal-Tutkun et al. suggested a useful diagnosis algorithm for BD uveitis based on ophthalmological criteria [54], although the results need to be validated in larger cohorts. The signs that provided the highest accuracy for the diagnosis of BD uveitis in patients with vitritis were the presence of retinitis foci, signs of occlusive retinal vasculitis, diffuse retinal capillary leakage on FA, and [55,56] the absence of granulomatous anterior uveitis or choroiditis (Table 2). Although a relapsing-remitting course has a high clinical value, this criterion was not relevant in this retrospective evaluation because patients were treated before spontaneous resolution [54]. Furthermore, the parafoveal microvasculature seems also to be affected in BD patients without uveitis [57,58,59,60]; likewise peripapillary microvascular changes could be detected by OCTA in BD patients without clinical ocular involvement [61]. FA is performed to ensure the absence of any vascular leakage or subclinical vasculitis [59]. OCTA appears to be promising in BD patients. Conventional color retinography and FA are limited in their field of view. Ultra-widefield imaging, which provides a 200° angle of photographic, autofluorographic, and angiographic views of the ocular fundus, has recently been introduced in ophthalmology. In the future, it is likely to become an essential tool in the diagnosis, treatment, and follow-up of retinal vasculitis, particularly those associated with BD. The laser flare meter can be used to monitor the degree of inflammation, as its values would correlate with the amount of vascular leakage visible on FA [62].

## 5. Treatment Modalities and Perspectives

### 5.1. BD Uveitis Management Recommendations

The goals of the therapeutic management of BD uveitis are to quickly and effectively control inflammation in order to preserve the visual function and limit irreversible structural damage, but also to treat the chronic subclinical inflammation, to prevent relapses and ocular complications, to limit ophthalmological and general adverse effects of iatrogenic causes, and to control systemic manifestations [63,64].

**Figure 2:**
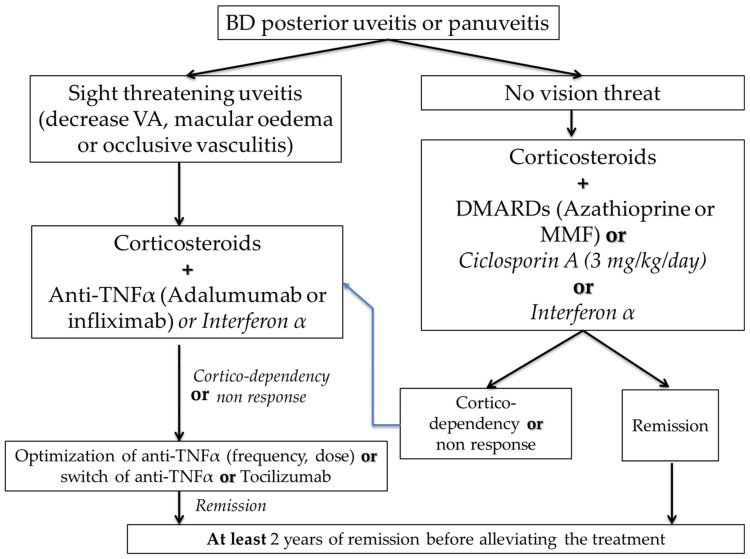
Management of uveitis in Behçet’s disease (extracted from the French recommendations for the management of Behçet’s disease. Kone-Paut, I. et al. [7]).

European and French recommendations on the treatment of BD were recently updated [7,65] (Figure 2). In the case of posterior segment ocular involvement, systemic immunosuppressive agents such as azathioprine, cyclosporine-A, interferon-α, and anti-TNF agents should be used with steroids. Patients presenting with sight-threatening uveitis should be treated with high-dose glucocorticoids and TNF inhibitors (intravenous infliximab (5 mg/kg), or subcutaneous adalimumab (80 mg then 40 mg/14 days) or interferon-α [7,66] as an option. Intravitreal corticosteroid injection could be a therapeutic option in patients with unilateral exacerbation as an adjunct to systemic treatment [7,65].

During BD uveitis management, a decrease in the immunomodulating treatment should be considered only after 2 years of remission and after steroids tapering to 5 mg daily or less. However, a study [67] has shown a high rate of relapse after the cessation of TNF inhibitors and suggest optimization by spacing the intervals between the doses [67]. The Biovas study [68] has recently shown lower relapse rate of uveitis with infliximab (5 mg/kg) every 4–6 weeks as compared to adalimumab 40 mg/14 days.

In the case of isolated AU, treatment is based on topical corticosteroids. However, systemic immunosuppressants such as azathioprine could be considered, in cases of risk factors of flares, such as young age, early onset of the disease, and male gender [7,66].

### 5.2. Screening for Relapse

Some recent studies have identified the criteria for predicting relapse and thus defining the point at which an escalation of therapy appears necessary to avoid visual impairment. A scoring system for determining the activity of ocular BD, termed Behçet’s disease ocular attack score 24 (BOS24) [69], has been used to predict visual acuity deterioration. BOS24 consists of a total 24 points divided into 6 parameters of ocular inflammatory symptoms [69] (Table 3). This score has different limitations and cannot replace the gold standard for the follow-up uveitis that is FA. In fact, diffuse capillary leakage on FA is an important supportive feature, because retinal vasculitis may not be readily apparent especially during the clinically quiescent periods [70,71]. A Thai study demonstrated that FA leakage, particularly of the optic disc and capillary vessels, after IFX therapy was strongly related to the presence of ocular inflammatory relapses in patients with ocular BD. FA is an important investigation for predicting poor visual outcome. Therefore, it should be carried out for every patient. However, BOS24 may also be a useful alternative when FA is unavailable, such as the limitation of time, cost, or machines [72] (Table 4). In fact, another Thai study showed that the BOS24 scoring system is an objective and quantitative measurement to evaluate the disease activity in patients with ocular Behçet’s disease, but further investigations and the accumulation of evidence are warranted to improve these scoring systems [73].

### 5.3. Peri- or Intraocular Treatment

Intravitreous corticosteroid infusions could be proposed as an adjuvant treatment in addition to systemic treatment for unilateral outbreaks. Intraocular pressure elevation and cataract development are the main side effects, in addition to the limited duration of action and lack of systemic disease control. This option can be used as a bridging therapy pending the escalation of therapy or, in rare cases, of absolute contraindication to some systemic therapies [75].

A first study of 15 patients with BD uveitis treated with intravitreal infliximab injections (1.5 mg intravitreal infliximab) showed a significant improvement in best-corrected visual acuity, with a significant reduction in macular thickness, retinal vasculitis, and retinitis [76]. Similarly, another study showed that intravitreal infliximab appeared to be safe and effective in the treatment of uveitis in 20 BD’s patients [77]. However, conflicting results regarding its safety and efficacy have been published in a study of 16 patients. Four eyes developed a severe immunological reaction and failure to control inflammation was described in the majority of eyes [78]. Intravitreal adalimumab was not successful in chronic refractory cystoid macular edema [79]. In this first study, no ocular or systemic adverse effects were observed. Subsequently, in the small population, intravitreal adalimumab was shown to be effective in controlling the inflammation, limiting uveitis flares, reducing macular edema, and improving the visual acuity in non-infectious uveitis including BD uveitis [55,56]. Nevertheless, there are conflicting results regarding the safety of intravitreal adalimumab infusions [79,80,81]. Further studies on the concentration and toxic effects of intravitreal injections anti-TNFα agents are needed, although the efficacy of these injections is not certain.

Intravitreal bevacizumab has been shown to be well tolerated and an effective adjunctive therapy in chronic uveitis, cystoid macular edema, and non-infectious uveitis particularly in BD; however, the median duration of effect was reduced [82].

Other molecules were developed in experimental autoimmune uveitis (EAU). Topical tacrolimus nano-capsule eye drops significantly reduced four typical inflammatory markers in a mouse model of keratitis, an inflammation of the anterior chamber [83]. In another register, significantly decreased progranulin expression was observed in patients with active BD. Progranulin (PGRN) is abundantly expressed in the immune cells, neurons, epithelial cells, and chondrocytes and plays a crucial role in several physiologic and pathologic processes including wound healing, neurodegeneration, tumorigenesis, and infection. Recently, PGRN has been reported to have anti-inflammatory functions. Recombinant PGRN significantly reduced EAU severity in association with a decreased frequency of Th17 and Th1 cells [84].

### 5.4. Conventional Immunosuppressants

Cyclosporine and azathioprine are the only two treatments that have been tested in randomized controlled trials (RCTs). In a large placebo-controlled trial, azathioprine (2.5 mg/kg per day) significantly decreased AU relapses and the development of new ocular disease after 2 years. None of the patients in the azathioprine group experienced serious adverse events, whereas one patient in the placebo group died of a pulmonary artery aneurysm [85]. Cyclosporin A was evaluated in three RCTs [86,87,88]. The response rates were between 80% and 91%, but safety was poor [86,87,88,89,90]. Cyclosporin A was significantly more effective than cyclophosphamide [91]. Nevertheless, nephrotoxicity limits its use in uveitis [92].

A longitudinal study using methotrexate (7.5–15 mg/week) showed an improvement or worsening of visual acuity in 46.5% and 37.2% of patients with BD uveitis, respectively [93].

Alkylant agents are not recommended due to their safety profile (malignancies and infertility) and the existing therapeutic alternative. In fact, biologics appear to be more effective and safer [92].

### 5.5. Interferons

Interferons are cytokines that can be synthesized by most cells and have antiviral, antiproliferative, and immunomodulatory functions. Their efficacy and tolerability have been analyzed in BD patients [94,95,96]. Several studies emphasized the efficacy and tolerance of IFN-α2a in patients with BD uveitis, in adults and pediatric BD patients [97,98,99,100,101,102,103]. Subcutaneous IFN-α2a (three million UI three times a week) is effective and safe for the long-term treatment of refractory BD uveitis. It allowed to reduce the duration of steroid treatment [104]. In addition, 90% of BD uveitis patients had a partial or complete response [92]. It would also allow, in some cases, long-term remission without treatment [40,105,106]. IFN-α2α was withdrawn from the market in 2020. Pegylated interferon-α-2a (PEG-IFN-α2a), given once a week, is still available. In one RCT, the addition of PEG-IFN-α2a to usual BD treatment with or without ocular involvement did not significantly reduce their cortico-dependence at 1 year. However, in those receiving corticosteroids at baseline, post hoc analysis demonstrated that the addition of PEG-IFN-α2a reduced the required corticosteroid dose with a significant improvement in quality of life [107]. A small case series has reported the efficacy of INF-α2b or INF-α2ain BD uveitis [108,109], even though INF-α2a was described to be more effective than INF-α2b [99]. Further studies are needed on the efficacy of the pegylated form efficacy in active disease and maintenance therapy of BD uveitis. The occurrence of influenza syndrome and mental disorders is the main limitation of interferon prescription [105]. Compared with anti-TNFα agents, this treatment does not promote serious infections, especially tuberculosis.

### 5.6. Anti-TNFα Agents

A retrospective study showed that anti-TNFα agents reached earlier ocular inflammation control and better steroid sparing than cDMARD in non-anterior non-infectious uveitis [110]. Infliximab and adalimumab are the two most used of the five anti-TNFα agents in BD uveitis.

Infliximab is a murine-human chimeric antibody against soluble and transmembrane forms of TNFα. The usual loading dose is 5 mg/kg given intravenously at weeks 0, 2, and 6, and then every 4 to 5 weeks [36,68]. In 158 patients, a rapid improvement in visual acuity and reduction in ocular inflammation were almost always reported, starting 24 h after infliximab treatment [111]. A significant reduction in uveitis flares was achieved in 89% of these patients. Based on these results, infliximab was approved in Japan for the treatment of “Behçet’s disease complicated by refractory uveoretinitis refractory to conventional therapies”. A prospective comparative study comparing relapses of acute panuveitis showed that infliximab (5 mg/kg), when given at the onset of uveitis, had a significantly faster effect in suppressing ocular inflammation than intravitreal triamcinolone (4 mg) or high-dose methylprednisolone (3-day course, 1 g/day) [112]. As control of acute ocular inflammation in BD is essential to prevent permanent vision loss, an intravenous infliximab should always be considered for panuveitis relapses in BD. No trials comparing infliximab and INF-α2a have been published but a meta-analysis showed similar remission rates with a higher sustained remission rate in the INF-α2a group (71%) compared to infliximab (43%). The rate of improvement visual acuity was 76% for infliximab and 46% for INF-α2a. Infliximab has a faster onset of action. The rate of discontinuation due to adverse effects was similar, i.e., 5.5% (INF-α2a group) vs 5% (infliximab group) [66].

Adalimumab is a fully human monoclonal antibody that binds TNFα, with the advantage of a subcutaneous form (80 mg followed by 40 mg every 2 weeks). Adalimumab was approved in 2016 for use in the treatment of non-infectious intermediate, posterior, and panuveitis. It was used first with success in case series [113,114,115], and then in several RCT studies [116,117,118]. The two RCTs vs placebo VISUAL I and VISUAL II evaluated the efficacy and safety of adalimumab in patients with active and inactive non-infectious uveitis of any cause, respectively [119,120]. A meta-analysis evaluated the efficacy and safety of anti-TNFα agents in the treatment of BD uveitis in 18 clinical trials, i.e., 15 retrospective studies and 3 prospective studies, from January 2010 to December 2019, with a minimum follow-up of 6 months and at least 10 patients with BD uveitis. The overall uveitis remission rate was 68% (95% CI 0.59–0.79), the visual acuity improvement rate was 60% (95% CI 0.47–0.77), the central macular thickness reduction was 112.70 μm (95% CI 72.8–153.0), with a significant corticosteroid-sparing effect. In this review, only 2.62% of the patients experienced serious adverse effects [121].

In the event of failure of a first anti-TNFα agent, switching to another may be useful. In a French multicenter study of 124 BD patients, 31 patients received a second line of anti-TNFα agent because of a lack of efficacy and/or side effects or because of the patients’ choice. In terms of ocular manifestations, complete and partial responses were observed in 12 (67%) and 5 (28%) patients, respectively [122]. An observational multicenter study compared the efficacy of infliximab versus adalimumab as a first-line treatment for refractory BD uveitis. In both groups (103 infliximab patients and 74 adalimumab patients), an improvement in all ocular parameters was observed after 1 year of therapy, with a significant difference in the improvement of anterior chamber inflammation, vitritis, and best-corrected visual acuity0 in the adalimumab group compared to the infliximab group. However, more rapid improvement in the anterior chamber inflammation and vitritis was observed with infliximab, even though patients in the adalimumab group did not receive a loading dose. The drug retention rate was higher in the adalimumab group (95.24% vs 84.95%; *p* = 0.042); 17.9% of infliximab and 14.9% of adalimumab patients discontinued treatment due to lack of efficacy. Interestingly, there was no significant difference between the two treatments in the improvement of vasculitis and macular edema [123].

The cumulative retention rate of adalimumab in 54 patients with BD uveitis at 12 and 48 months of follow-up was 76.9% and 63.5%, respectively. It was not influenced by the concomitant use of DMARDs or by the different lines of biologic agents. In addition, the retention rate was not reduced in patients with known negative prognostic factors for BD ocular involvement, such as male gender, early age at disease onset, and the duration of uveitis. Similarly, the cumulative retention rates of infliximab in 40 patients with BD uveitis at 12, 24, 60, and 120 months of follow-up were 89.03%, 86.16%, 75.66%, and 47.11%, respectively, and were not modified by the use of concomitant DMARDs or by known negative prognostic factors. A significantly lower discontinuation rate was observed when infliximab was administered after other biologics. At 10-year follow-up, discontinuation was due to: secondary failure (six patients), primary failure (two patients), adverse events (four patients), prolonged disease remission (two patients), and switching to subcutaneous treatment (one patient) [124]. A Japanese team showed that IFX monotherapy was effective and not inferior to combination therapies such as colchicine or corticosteroids in refractory BD uveitis over a 10-year follow-up period [125].

If ineffective, the increasing the dose of infliximab or reducing the frequency of administration has been described in the treatment of BD uveitis [123]. Similarly, reducing the dose of adalimumab to weekly has recently been described in uveitis with encouraging results, but not specifically in this indication [126,127].

Etanercept is a fusion protein, which is a soluble receptor that binds to soluble TNFα and prevents it from binding to target cells. There are substantial data suggesting that etanercept is less effective than anti-TNFα antibodies in the treatment of uveitis [128].

Golimumab is a fully human anti-TNFα monoclonal antibody. The constant regions of the heavy and light chains of golimumab are identical in amino acid sequences to those of infliximab. Several case reports and series have demonstrated the successful control of severe uveitis with golimumab, particularly in juvenile idiopathic arthritis and BD [129,130,131,132,133].

Certolizumab *pegol* is a pegylated recombinant humanized antibody Fab fragment against TNFα. The pegylation of the antibody delays clearance. The reported experience of using certolizumab-pegol for the treatment of BD uveitis is currently limited.

Anti-TNFα agents are associated with a specific increased risk of tuberculosis (TB) [134]. Screening for latent TB and prophylactic anti-TB treatment for all those found positive is recommended for all patients planning to start therapy with anti-TNFα agents. Patients receiving anti-TNFα agents may develop a variety of serious opportunistic infections, particularly those involving intracellular microorganisms. Several demyelinating and neurological events, including exacerbations of pre-existing multiple sclerosis, have been reported in patients receiving anti-TNFα agents [135,136]. There is no conclusive evidence of an increased risk of solid tumors or lymphoproliferative disorders with anti-TNFα agents [135,136], except for non-melanoma skin cancer [137]. All anti-TNFα agents can induce antinuclear antibodies, but the development of anti-TNFα-induced lupus is less commonly reported. Local complications at the site of drug administration have been frequently reported. Anti-TNFα agents may induce the formation of neutralizing antibodies, resulting in loss of efficacy and the occurrence of infusion reactions [138]. New onset and the worsening of congestive heart failure have been reported [135]. A rare and paradoxical adverse event is the development of sarcoidosis during anti-TNFα therapy, as well as the paradoxical occurrence of psoriasis [128].

### 5.7. Biologics beyond the Anti-TNFα Agents

#### 5.7.1. Anti-Interleukin-6 Agents

Tocilizumab (TCZ) is a humanized anti-interleukin-6 (IL-6) receptor monoclonal antibody that inhibits the IL-6 pathway by preventing IL-6 from binding to its receptor. In the prospective STOP-Uveitis study on 37 patients, intravenous TCZ was found to be safe and equally effective in both naïve and previously treated patients with non-anterior non-infectious uveitis, mostly idiopathic, including for the case of a BD uveitis [139]. In a retrospective study, TCZ was shown to be effective in 5 cases of BD uveitis refractory to IFN-α and anti-TNFα agents, when administered intravenously at 8 mg/kg [140], and in 11 cases of BD uveitis refractory to anti-TNFα agents [141]. A recent review of the literature, which aimed to summarize the original articles published on PubMed and EMBASE up to December 2021 reporting on the use of tocilizumab in BD, showed that tocilizumab was effective in 87% of anti-TNF-naïve patients and in 80% of anti-TNF-experienced patients in 25 articles involving a total of 74 patients [142]. Moreover, a recent retrospective multicenter French study, aimed at analyzing the factors associated with response to anti-TNFα agents and tocilizumab in patients with 69 refractory uveitic macular edema, showed in multivariate analysis that treatment with tocilizumab (OR 2.10 [95% CI 1.06–4.06], *p* = 0.03) was independently associated with a complete response of uveitic macular edema, compared to anti-TNFα agents, so, tocilizumab seems to improve complete response of uveitic macular edema compared to anti-TNFα agents [36].

#### 5.7.2. Anti-Interleukin-1 Agents

Three anti-interleukin-1 (IL-1) agents have been studied in BD treatment: anakinra, an IL-1 receptor antagonist protein, canakinumab, a human anti-IL-1β monoclonal antibody, and the recombinant humanized anti-IL-1β gevokizumab. Their place in the treatment of BD uveitis remains unclear, due to conflicting results in the literature. In a randomized, double-masked, placebo-controlled trial in patients with BD uveitis who had experienced an ocular exacerbation, gevokizumab did not significantly reduce the median time to relapse [143]. However, in an open-label study, a single infusion of gevokizumab resulted in a rapid and sustained reduction in intraocular inflammation in seven patients with resistant BD uveitis [144]. Anakinra controlled ocular inflammation in three out of four BD uveitis patients. However, patients relapse over time [145]. In a case series, of four patients with recurrent BD uveitis refractory to anti-TNFα agents, three showed a complete resolution of ocular inflammation with anakinra, but a relapse of uveitis occurred after a mean of 24 weeks [145]. In a retrospective multicenter study, anakinra and canakinumab were shown to be effective and safe in 73% out of 30 patients, including 16 with BD uveitis. The most common adverse events were local skin reactions [146]. In an observational study, anakinra or canakinumab were evaluated in 19 BD uveitis and improved retinal vasculitis and reduced the rate of uveitis flares. However, no significant effect in macular thickness or visual acuity was observed [75].

#### 5.7.3. Anti-Interleukin-17 Agents

Secukinumab, the only anti-IL-17 agent studied in the treatment of uveitis, is a fully human monoclonal antibody [147]. It has been tested subcutaneously against placebo in three RCTs. The SHIELD study included 118 patients with non-anterior BD uveitis, the INSURE study analyzed 31 patients with non-anterior non-BD uveitis, and the ENDURE study analyzed 125 patients with quiescent non-anterior non-BD uveitis. In the SHIELD study, as in the other two studies, the primary endpoint of a reduction in the rate of uveitis recurrence was not met. The secondary efficacy data from SHIELD and INSURE may suggest a potential beneficial effect of secukinumab in reducing the use of concomitant immunosuppressants [148].

Nevertheless, a prospective study suggested the efficacy of intravenous secukinumab in the treatment of active chronic non-infectious uveitis requiring systemic immunosuppression in 16 patients, including one with BD uveitis [149]. Similarly, a subsequent prospective study reported that intravenous secukinumab was more effective and better tolerated than subcutaneous secukinumab in 37 patients without BD uveitis, in patients with non-infectious uveitis requiring systemic corticosteroid-sparing immunosuppressive therapy [150].

A retrospective multicenter study in 15 patients with BD refractory to colchicine, DMARDs, and at least one anti-TNFα agent reported the efficacy and safety of secukinumab in the treatment of mucosal and articular manifestations. One patient with active anterior uveitis at the time of initiation of secukinumab did not experience an ocular flare during follow-up [151].

#### 5.7.4. Anti-Interleukin-12/23 Agents

Ustekinumab is a fully humanized monoclonal antibody with a high affinity for the common p40 subunit of IL-12 and IL-23, which appears to play a critical role in non-infectious uveitis [152]. Efficacy data in BD uveitis are not yet available.

#### 5.7.5. Other Biologics

Rituximab is a B-cell targeted therapy. Davatchi et al. reported the efficacy of rituximab in combination with methotrexate vs a combination of pulse cyclophosphamide and azathioprine in BD uveitis with no significant differences between the two groups [153].

Alemtuzumab is a humanized anti-CD52 monoclonal antibody. A single infusion of alemtuzumab was given to 18 patients including five BD patients with ocular involvement, all of whom were in complete or partial remission, at 6 months [154]. In a retrospective study, 21 patients out of 32 BD patients had ocular involvement and all of them achieved remission [155].

Abatacept is a T-cell targeted therapy, capable of blocking CD-80 and CD-86 on antigen-presenting cells, which are necessary for their activation. Short-term efficacy has been described in a case report of refractory BD-associated scleritis [156].

Daclizumab, a humanized monoclonal antibody that binds CD25 of the IL-2 receptors, was studied in a randomized, placebo-controlled trial in 17 BD patients, it was not superior to placebo in preventing relapses and tapering immunosuppressive drugs [157]. It was withdrawn from the market in 2018 after reports of autoimmune encephalitis [158].

#### 5.7.6. Targeted Synthetic Disease-Modifying Antirheumatic Drugs

Targeted synthetic disease-modifying antirheumatic drugs (tsDMARDs) include phosphodiesterase inhibitors and kinase inhibitors. Their small size gives them a high level of bioavailability, and moreover tsDMARDs also have a low rate of immunogenicity [159].

Tofacitinib is an anti-Janus kinase (JAK) 1/3 inhibitor. A study in 13 patients with refractory BD suggested its safety and potential efficacy in vascular and joint involvement. No ocular involvement was described [160]. In a case series of two patients, tofacitinib was interesting for refractory, non-infectious idiopathic uveitis or scleritis [161]. Encouraging results have recently been reported in uveitis associated with juvenile idiopathic arthritis [162].

Apremilast, a phosphodiesterase 4 inhibitor, modulates cytokines that are upregulated in BD. Its efficacy has been demonstrated in phase 2 and 3 randomized, placebo-controlled clinical trials in BD oral ulcers and it is now approved for this indication. However, its potential role in the treatment of BD uveitis has not yet been investigated [163,164,165].

## 6. Conclusions

Despite diagnostic and therapeutic innovations, BD uveitis remains severe. Clinicians need to be aware of the criteria for uveitis in relation to BD. Improvements in multimodal ocular imaging are likely to improve the assessment of patients. However, there are still cases of BD uveitis that are refractory to the recommended treatment and studies comparing the various existing biologics will help to improve management. The therapeutic armamentarium is expanding and alternatives to anti-TNFα or interferon, especially anti-IL-6 agents, are likely to be useful in refractory macular edema. Some questions remain, such as the duration of treatment. Clinicians should pay attention to FA leakage, particularly of the optic disc and capillaries or evaluate BOS24 if FA is not available, when following patients with ocular BD after the initiation of systemic treatment to define the best time for therapeutic escalation and avoid visual impairment and improve prognosis.

## Figures and Tables

**Table 1:** International criteria for Behçet’s disease (adapted from The International Criteria for Behçet’s Disease (ICBD): a collaborative study of 27 countries on the sensitivity and specificity of the new criteria [39]).

Sign/Symptom	Points
Ocular lesions	2
Genital aphtosis	2
Oral aphtosis	2
Skin lesions	1
Neurological symptoms	1
Vascular manifestations	1
Positive pathergy test ^1^	1

^1^ The pathergy test is optional and the primary scoring system does not include pathergy testing. However, where pathergy testing is conducted, one extra point may be assigned for a positive result. Diagnostic of Behçet’s disease if score > 4.

**Table 2:** Criteria pointing to uveitis in relation to Behçet’s disease (extracted from Uveitis in Behçet disease: an analysis of 880 patients, Tugal-Tuknun I. et al. [13] and An Algorithm for the Diagnosis of Behçet Disease Uveitis in Adults, Tugal-Tuknun I. et al. [54]).

**1.** **Demography**
Male patient
Mean age at onset of the uveitis: 28.5–30 years old
Originated from Mediterranean basin, the Middle East, and Asia
**2.** **Characteristics of uveitis nature**
Bilateral uveitis
Rarely isolated anterior uveitis (<10%)
Recurrent flares
Posterior uveitis (with retinal vasculitis or its sequelae and/or retinal infiltrate) or panuveitis
Presence of retinal nerve fiber layer defect
Presence of macular edema (the most common complication)
Presence of diffuse capillary leakage on fluorescein angiography
Association with peripheral occlusive periphlebitis or gliotic sheathing or ghost vessels
Association with retinal vein branch occlusion
Negative signs:
Non granulomatous uveitis
Not associated with choroiditis
**3.** **Extraophthalmological associated signs of BD**
Recurrent oral ulcers, genital aphtosis
Pseudofolliculitis, erythema nodosa
Neurological symptoms
Vascular manifestations
Positive pathergy test

**Table 3:** BOS24 scoring (Reprinted from Behçet’s disease ocular attack score 24: evaluation of ocular disease activity before and after initiation of infliximab by Toshikatsu Kaburaki et al. [69]).

**(1) Cells in the anterior chamber (max. 4 points)**
Cell 0: 0 point, cell 0.5+ or 1+ : 1 point, cell 2+ : 2 points, cell 3+ : 3 points, cell 4+ or hypopyon: 4 points
**(2) Vitreous haze (max. 4 points)**
Haze 0: 0 point, haze 0.5+ or 1+ : 1 point, haze 2+ : 2 points, haze 3+ : 3 points, haze 4+ : 4 points
**(3) New inflammatory changes in the peripheral retina (max. 8 points)**
Give each 2 points in each quadrant of the peripheral retina if new inflammatory changes (exudates, hemorrhages, vasculitis) are seen
**(4) New inflammatory changes in the posterior pole of retina (max. 4 points)**
0%: 0 point, > 0 and < 10%: 2 points, ≥ 10 and < 25%: 3 points, ≥ 25%: 4 points
**(5) New inflammatory changes in the fovea (max. 2 points)**
Give 2 points if new inflammatory changes (exudates, hemorrhages, vasculitis) are seen in the fovea
**(6) New inflammatory changes in the optic disc (max. 2 points)**
Give 2 points if new inflammatory changes in the optic disc (redness and edema, sometimes accompanied by hemorrhages, exudates and edema of retina surrounding the optic disc) are seen

**Table 4:** Factors linked to Behçet’s uveitis relapse and should lead to therapeutic escalation (Extracted from: Fluorescein angiographic findings and Behcet’s disease ocular attack score 24 (BOS24) as prognostic factors for visual outcome in patients with ocular Behcet’s disease [72] and The Relationship between Fluorescein Angiography Leakage after Infliximab Therapy and Relapse of Ocular Inflammatory Attacks in Ocular Behcet’s Disease Patients [74]).

**1. Fundus fluorecesin angiography parameters**
Severe posterior pole leakage particularly on the optic disc and capillary vessels
Vitreous haze
Arterial narrowing
**2. If FA is unvailable**
BOS24 ≥ 6

## Data Availability

No new data were created.
